# Heritability of female extra-pair paternity rate in song sparrows (*Melospiza melodia*)

**DOI:** 10.1098/rspb.2010.1704

**Published:** 2010-10-27

**Authors:** Jane M. Reid, Peter Arcese, Rebecca J. Sardell, Lukas F. Keller

**Affiliations:** 1 Institute of Biological and Environmental Sciences, School of Biological Sciences, University of Aberdeen, Zoology Building, Tillydrone Avenue, Aberdeen AB24 2TZ, UK; 2 Centre for Applied Conservation Research, Department of Forest Sciences, University of British Columbia, 2424 Main Mall, Vancouver, British Columbia, CanadaV6T 1Z4; 3 Institute of Evolutionary Biology and Environmental Studies, University of Zurich, Winterthurerstrasse 190, 8057 Zurich, Switzerland

**Keywords:** animal model, good genes, mate choice, polyandry, sexual selection

## Abstract

The forces driving the evolution of extra-pair reproduction in socially monogamous animals remain widely debated and unresolved. One key hypothesis is that female extra-pair reproduction evolves through indirect genetic benefits, reflecting increased additive genetic value of extra-pair offspring. Such evolution requires that a female's propensity to produce offspring that are sired by an extra-pair male is heritable. However, additive genetic variance and heritability in female extra-pair paternity (EPP) rate have not been quantified, precluding accurate estimation of the force of indirect selection. Sixteen years of comprehensive paternity and pedigree data from socially monogamous but genetically polygynandrous song sparrows (*Melospiza melodia*) showed significant additive genetic variance and heritability in the proportion of a female's offspring that was sired by an extra-pair male, constituting major components of the genetic architecture required for extra-pair reproduction to evolve through indirect additive genetic benefits. However, estimated heritabilities were moderately small (0.12 and 0.18 on the observed and underlying latent scales, respectively). The force of selection on extra-pair reproduction through indirect additive genetic benefits may consequently be relatively weak. However, the additive genetic variance and non-zero heritability observed in female EPP rate allow for multiple further genetic mechanisms to drive and constrain mating system evolution.

## 1. Introduction

Molecular genetic analyses have revealed that socially monogamous populations are frequently genetically polygynandrous, undermining previously accepted views of animal mating systems and requiring new theories explaining mating system evolution [1–3]. The evolution of extra-pair reproduction by socially monogamous males can be relatively easily understood if extra-pair paternity (EPP) directly increases an individual male's reproductive success and hence fitness [4]. However, the forces driving extra-pair reproduction by socially monogamous females remain unclear and widely debated [1–3,5–7]. Since extra-pair mating does not necessarily increase a female's immediate reproductive success, any direct fitness benefits are often less obvious than the potential direct costs (such as sexually transmitted disease and reduced paternal care by the female's cuckolded social mate [1,3,4]). Any pro-active extra-pair reproduction by females is therefore widely hypothesized to reflect indirect additive or non-additive genetic benefits that increase offspring fitness [1,2,5–9].

Consequently, numerous empirical studies have probed the possible indirect benefits of female extra-pair reproduction by relating EPP to male secondary sexual ornamentation and measures of offspring condition, fitness and genetic heterozygosity [2,3,6,7,10–13]. However, as with all hypotheses of mating system evolution through indirect genetic benefits, rigorous tests ideally require explicit estimation of key genetic and phenotypic variances and covariances, and hence explicit estimation of indirect components of selection [5,14–18].

In the specific context of explaining the evolution of female extra-pair reproduction, an expression describing the force of selection through indirect additive genetic benefits has been derived as1.1
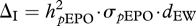
where *Δ*_I_ is the number of phenotypic standard deviations by which the mean EPP rate would evolve in one generation through such indirect selection alone, 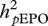 is the heritability of the proportion of a female's offspring that is sired by an extra-pair male (*p*EPO), *σ*_*p*EPO_ is the phenotypic standard deviation of this proportion and *d*_EW_ is the within-brood difference in mean (additive genetic) fitness between extra-pair offspring (EPO) and within-pair offspring (WPO) [5]. This expression is derived from a more general expression describing the evolution of female preferences [14]. Arnqvist & Kirkpatrick [5] describe key quantities in terms of female extra-pair copulation (EPC) rate rather than EPP rate, prompting debate as to whether selection on EPCs or EPP is, or should be, considered [4]. However, in fact they derive *Δ*_I_ in terms of the proportion of a female's offspring that is sired by an extra-pair male ([5], their appendix). Indeed, it is EPP rather than EPC rate that is most directly relevant in the specific context of estimating indirect selection on female extra-pair reproduction, since EPCs that do not result in EPO cannot create linkage disequilibrium between genes promoting female extra-pair reproduction and those contributing to high fitness, or therefore cause indirect selection on a female's tendency to produce EPO (§4). One route to testing the specific hypothesis that female extra-pair reproduction at least partly reflects indirect additive genetic benefits is therefore to estimate 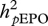, *σ*_*p*EPO_ and *d*_EW_ and hence the magnitude of *Δ*_I_ [5].

Several empirical studies have attempted to estimate the difference in phenotypic fitness between WPO and EPO [5,6,13]. However, no studies have yet measured and compared overall fitness, as opposed to fitness components or traits that are hypothesized to be correlated with fitness (e.g. [11,13,19]). Furthermore, strictly, *d*_EW_ is the regression of the genetic component of fitness that offspring inherit from males on a female's propensity for extra-pair reproduction [5]. It therefore equals the within-brood difference in paternal additive genetic value for fitness between WPO and EPO (assuming equal average environmental and maternal effects), not the difference in phenotypic fitness. No empirical studies have attempted to estimate this specific quantity. Estimating *σ*_*p*EPO_ is straightforward given data describing *p*EPO for all females in a population, although empirical estimates have not in fact been reported. However, a maximum can be calculated as *σ*_*p*EPO,max_ = √(*μ*_EPP_(1 − *μ*_EPP_)), where *μ*_EPP_ is the mean population-wide EPP rate [5]. This information is readily available (e.g. [2,20]). Finally, the heritability 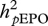, defined as the proportion of total phenotypic variance in the proportion of a female's offspring that is sired by an extra-pair male that is attributable to additive genetic variance (*V*_A,*p*EPO_), has not been rigorously quantified in any natural or laboratory population. The only available data concern remating propensity (sequential polyandry) rather than simultaneous polyandry, and relate to tightly controlled laboratory invertebrate populations with restricted variation in mating opportunity [18]. Published estimates of *Δ*_I_ have consequently assumed 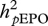 = 1.0 or 0.4, thereby setting maximum possible or probable magnitudes of *Δ*_I_ given estimated or postulated *d*_EW_ and *σ*_*p*EPO_ [5]. However, in reality, 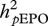 is extremely unlikely to approach 1.0. Although estimated heritabilities of mating behaviours and preferences can be high (≥0.4), they are not always so [21,22]. Heritabilities of life-history traits and fitness components are often small, reflecting low additive genetic variance (*V*_A_) and/or high residual variance [23–25]. Since 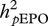, *σ*_*p*EPO_ and *d*_EW_ contribute multiplicatively to *Δ*_I_ (equation (1.1)), evidence that 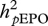 is small or zero would render explicit estimation of *d*_EW_ rather redundant in the context of testing the specific hypothesis that female extra-pair reproduction reflects indirect additive genetic benefits. Quantifying *V*_A,*p*EPO_ and 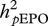 in socially monogamous animals experiencing natural variation in mating and reproductive success is therefore central to testing key hypotheses explaining extra-pair reproduction.

Estimating *V*_A,*p*EPO_ and hence 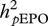 in free-living animals requires data describing the within-pair and extra-pair reproductive success of many females of known relatedness. We used 16 years of comprehensive reproductive success, paternity and pedigree data to estimate *V*_A,*p*EPO_ and hence 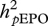 in free-living song sparrows (*Melospiza melodia*), and thereby consider the possible role of indirect additive genetic benefits in driving the evolution of female extra-pair reproduction.

## 2. Material and methods

### (a) Study system

A resident population of song sparrows inhabiting Mandarte Island, British Columbia, Canada, recently numbering 11–49 breeding pairs, is well suited to such analyses. Since 1975, all territories and breeding attempts have been closely monitored, all clutch and brood sizes have been recorded and all offspring surviving to *ca* 6 days post-hatch have been colour-ringed before leaving their natal territory [26]. All immigrants to Mandarte (1.1 per year on average) have been caught and colour-ringed soon after arriving. All population members are therefore individually identifiable by resighting [26]. In all years, all social pairings and thus the social parents of all offspring (those incubating clutches and provisioning chicks) were identified, except that some offspring fledged in 1980 had unknown parents owing to reduced fieldwork in that year [26,27]. Female song sparrows typically breed two to three times per year, lay three or four eggs per clutch and do not always remain paired to the same social mate across different breeding attempts or years [26,27]. Immigration is sufficient to maintain genetic diversity and prevent inbreeding from accumulating [28]. There is evidence of additive genetic variance (*V*_A_) and substantial inbreeding depression in fitness components [27,29].

### (b) Paternity analyses

During 1993–2008, 99.4 per cent of all ringed offspring and their parents were blood-sampled and genotyped at 13 polymorphic microsatellite loci [20]. These genetic data were used to identify WPO that were sired by a female's socially paired male and EPO that were sired by males to whom a female was not currently socially paired [20]. Cuckolded males were excluded as sire with probability ≈0.9998. Genetic mothers and fathers were assigned to all chicks using Bayesian full probability models that utilized genetic and spatial information [20,30]. These analyses suggested that all mothers were correctly identified by social behaviour, and assigned genetic fathers with high confidence. In summary, sires of 99.2 per cent (2189/2207) of blood-sampled offspring were assigned with 95 per cent or more individual-level confidence. Sires were assigned with less than 80 per cent individual-level confidence for only 0.2 per cent (5/2207) offspring, and the number of unsampled sires in the population (estimated within the paternity analysis) was approximately zero [20]. Overall, 627 of 2207 (28.4%) offspring were identified as EPO [20]. The mean EPP rate (*μ*_EPP_) of *ca* 28 per cent was therefore relatively similar to that observed in a nearby mainland song sparrow population (24%), and not remarkable compared with rates observed in birds more widely [2,20]. The annual proportion of each female's offspring that was sired by an extra-pair male was then calculated as *p*EPO = *n*EPO/(*n*EPO + *n*WPO), where *n*EPO and *n*WPO are the numbers of EPO and WPO that a female reared to ringing within a single year. In total, *p*EPO was measured for 204 individual females that reared one or more offspring to ringing in 1 year or more during 1993–2008.

### (c) Statistical analyses

We first estimated the among-individual variance in *p*EPO (*V*_I_), and hence the repeatability of *p*EPO (*R*_*p*EPO_), by fitting a generalized linear mixed model (GLMM) with random effects of individual females, binomial error structure and *n*EPO and (*n*EPO + *n*WPO) as the binomial numerator and denominator, respectively. To determine whether observed repeatability reflected additive genetic variance, we estimated *V*_A,*p*EPO_ using a GLMM where pairwise coefficients of kinship (*k*) among individuals defined a matrix proportional to the variance–covariance structure of additive genetic random effects (an ‘animal model’ [31–33]). Random effects of individual females were retained so that permanent (‘environmental’) variance associated with an individual (*V*_PI_), and residual variance (*V*_R_), was also estimated within the animal model.

Accurate estimation of *V*_A,*p*EPO_ and hence 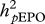 using this method requires accurate pedigree data linking all individuals with observed phenotypes and their ancestors (hence allowing accurate estimation of *k*). We used all available behavioural data to compile a pedigree linking all adult sparrows that had hatched on Mandarte during 1975–2008 to their observed social mother and father [27,34]. The genetic paternity assignments were then used to correct the pedigree paternity of individuals hatched during 1993–2008 to their most likely true sire. Since 0/18 blood-sampled offspring whose sires were assigned with less than 95 per cent individual-level confidence and zero unsampled offspring hatched during 1993–2008 survived to adulthood, the relatively high paternity uncertainty in these cases caused no pedigree error. The pedigree data covering adult sparrows that had hatched during 1993–2008 were therefore complete and highly resolved, with no gaps and greater than 95 per cent statistical confidence in all individual links. The pre-1993 pedigree data still contain error owing to unobserved EPP during 1975–1992. However, assuming *μ*_EPP_ ≈ 0.28 and no error in maternity (as observed during 1993–2008), *ca* 86 per cent of all pre-1993 pedigree links will be correct. Estimates of *k* among females breeding in 1993 calculated from the pre-1993 data are therefore highly informative, and likely to be more biologically relevant than an assumption of zero relatedness [29]. We therefore used all available pedigree data, pruned to the females whose *p*EPO was measured and all known ancestors, to estimate the *k* matrix. In practice, results remained quantitatively similar when analyses were repeated using only the corrected 1993–2008 pedigree data. Since microsatellite genotypes suggest that immigrants are not closely related to existing Mandarte natives, *k* between new immigrants and natives was defined as zero [28,34].

Animal models can return inflated estimates of *V*_A_ if additional sources of phenotypic covariance among relatives, such as common brood, territory or maternal environmental effects, are not adequately modelled [32]. Since the 204 females for which *p*EPO was measured fledged from 189 different broods produced by 117 different mothers across 156 different mother-years, there was limited potential for common brood effects to inflate estimates of *V*_A,*p*EPO_ and limited power to estimate maternal environmental variance. However, to verify whether territory or maternal effects could have inflated *V*_A,*p*EPO_, we re-ran models with random effects of territory and mother identity [32]. Territory and maternal variances were estimated as *ca* zero, and estimates of *V*_A,*p*EPO_ and 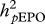 remained quantitatively similar whether or not these variance components were included in the model. Estimates of *V*_A,*p*EPO_ and 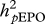 also remained similar when analyses were rerun using data from one randomly selected female per brood and per mother. Finally, since estimates of *V*_A_ may be inflated by unmodelled inbreeding depression [35,36], we fitted a fixed regression on individual coefficient of inbreeding (*f*) within the animal model, thereby additionally providing an estimate of inbreeding depression in female *p*EPO. Inbreeding coefficients were calculated relative to the 1975 pedigree baseline using standard algorithms [27,34].

### (d) Analysis implementation

Since animal models for non-Gaussian traits can be challenging to fit using maximum likelihood [37,38], we used Bayesian methods and estimated the posterior mode and 95 per cent credible intervals (95% CI) for fixed effects, variance components and heritabilities assuming binomial errors, logit link and additive overdispersion. Exploratory analyses suggested that overall EPP rates varied among years but did not vary markedly with female age. All models therefore included fixed effects of year but not age.

The repeatability and heritability of *p*EPO were estimated on the latent (logit) scale as *R*_*p*EPO,lat_ = *V*_I_/(*V*_I_ + *V*_R_ + *π*^2^/3) and 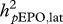 = *V*_A,*p*EPO_/(*V*_A,*p*EPO_ + *V*_PI_ + *V*_R_ + *π*^2^/3), respectively (since the logistic variance is proportional to *π*^2^/3 [39]). 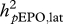 is interpretable as the genetic intra-class correlation (the expected correlation of *p*EPO on the logit scale between monozygotic twins), or as the heritability of a latent variable describing a female's underlying propensity to produce EPO [31,39]. The observed data-scale repeatability and heritability, describing the proportion of a female's offspring that were EPO, can be estimated as *R*_*p*EPO,obs_ = (*V*_I_*X*^2^/(1 + *μ*_EPP_)^2^)/((*V*_I_ + *V*_R_)*X*^2^)/(1 + *μ*_EPP_)^2^ + *X*(1 − *X*)) and 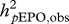 = (*V*_A,*p*EPO_*X*^2^/(1 + *μ*_EPP_)^2^)/((*V*_A,*p*EPO_ + *V*_PI_ + *V*_R_)X^2^)/(1 + *μ*_EPP_)^2^ + *X*(1 − *X*)), where *X* = *μ*_EPP_/(1 + *μ*_EPP_) [39]. Estimates of 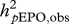 are therefore not independent of the mean observed EPP rate (*μ*_EPP_), and consequently cannot be readily compared across environments or populations [31,40]. However, we estimated 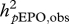 as well as 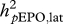 to allow population-specific parameterization of equation (1.1).

Models were fitted in R v. 2.10.1 using library MCMCglmm [41,42] with 3 005 000 iterations, burn-in 5000 and thinning interval 3000. Autocorrelation among consecutive observations was low (*r* < 0.05). Fixed effects priors were normally distributed and diffuse with mean 0 and variance 10^8^. Parameter-expanded random effects priors were vague and proper, with normally distributed working parameter priors with mean 0 and variance 625 and inverse-Wishart distributed location effect priors with degree of belief parameter and limit variance of 1. Conclusions were robust to substantial variation in prior specifications. Analyses of a simulated null trait returned *V*_A_ and *h*^2^ ≈ 0 (as expected since the 95% CI for a variance component cannot overlap zero). Data for immigrant females were excluded because sample sizes were insufficient to estimate effects of immigrant status on *p*EPO and because *f* is undefined for immigrants (as opposed to their offspring [34]). Female *p*EPO was estimated per year rather than per brood to reduce any correlation with sire or social pair effects and provide a parallel trait to male extra-pair reproductive success (which cannot be estimated on a per-brood basis [29]). There was no consistent among-female variation in the total number of offspring ringed per year (*n*EPO + *n*WPO, posterior mode for *V*_I_: <0.001, 95% CI: <0.0001–0.01). The estimated proportion of offspring that were EPO was not correlated with the total offspring ringed per female per year (posterior correlation: 0.01, 95% CI: −0.09–0.15). A coefficient of additive genetic variance (CV_A_) for *p*EPO was not calculated because *V*_A,*p*EPO_ was estimated on transformed scales [23].

Finally, to allow parametrization of equation (1.1), we estimated the phenotypic standard deviation of *p*EPO (*σ*_*p*EPO_) across all observations and within each year, and calculated the maximum possible standard deviation (*σ*_*p*EPO,max_) as √(*μ*_EPP_(1 − *μ*_EPP_)).

## 3. Results

### (a) Phenotypic variation and repeatability in *p*EPO

The proportion of a female's offspring that was sired by an extra-pair male (*p*EPO) was measured for 204 individual females that reared one or more offspring within a particular year, comprising 416 female-years in total. Overall *σ*_*p*EPO_ was 0.32 (ranging from 0.22 to 0.39 in individual years). *σ*_*p*EPO,max_ was 0.45 given *μ*_EPP_ = 0.284.

**Figure 1.**
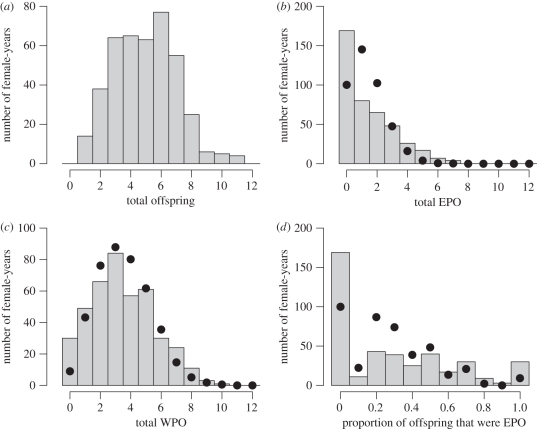
Distributions of the annual number of (*a*) offspring ringed, (*b*) extra-pair offspring (EPO) ringed, (*c*) within-pair offspring (WPO) ringed and (*d*) the proportion of offspring ringed per year that were EPO (*p*EPO) observed across all females that reared one or more offspring to ringing in a particular year (bars) and expected given constant and uniform *p*EPO (circles). Expected frequencies were estimated by simulation given the observed total offspring ringed per female per year and a mean extra-pair paternity rate of *μ*_EPP_ = 0.284.

Across all 416 observations, *p*EPO showed substantial extra-binomial variance, demonstrating variation in the underlying probability of producing an EPO (figure 1, posterior mode for latent-scale residual variance *V*_R_: 2.8, 95% CI: 2.2–4.1). Furthermore, a GLMM with random effects of individual females demonstrated substantial among-female variation (posterior modes: *V*_I_: 1.03, 95% CI: 0.44–1.92; *V*_R_: 1.89, 95% CI: 1.17–2.80). *p*EPO was therefore moderately repeatable within individual females across years on the latent (logit) scale describing a female's underlying liability to produce EPO rather than WPO (posterior mode for *R*_*p*EPO,lat_: 0.19, 95% CI: 0.08–0.28) and on the observed scale (posterior mode for *R*_*p*EPO,obs_: 0.13, 95% CI: 0.06–0.21). Estimated repeatabilities remained quantitatively similar when analyses were restricted to females with more than one observation.

### (b) Additive genetic variance and heritability in *p*EPO

The pruned pedigree contained 455 individuals. Mean *k* among the 204 females whose *p*EPO was measured was 0.072 (median 0.064, range 0.005–0.409). The animal model estimated substantial *V*_A,*p*EPO_, implying substantial additive genetic variance in a female's liability to produce EPO rather than WPO (table 1). Furthermore, despite substantial residual variance, the posterior mode for latent-scale 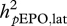 was 0.18 and the 95% CI did not converge to zero (table 1). The posterior mode for data scale 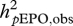 was 0.12, and also exceeded zero (table 1). The permanent individual variance (*V*_PI_) was close to zero (table 1), suggesting that most repeatable among-female variation in *p*EPO was additive genetic. Indeed, *R*_*p*EPO_ exceeded 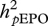 by less than 5 per cent on both latent and observed scales (table 1).

**Table 1.** Posterior modes (and 95% CI) for variance components, latent-scale heritability, observed data-scale heritability and inbreeding depression in the annual proportion of a female's offspring that was sired by an extra-pair male (*p*EPO). The data-scale heritability was estimated assuming *μ*_EPP_ = 0.284.

additive genetic variance (*V*_A_)	permanent individual variance (*V*_PI_)	residual variance (*V*_R_)	latent-scale heritability ( 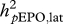 )	data-scale heritability ( 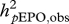 )	inbreeding depression (*β*_f_)
1.08 (0.16–2.18)	0.005 (<0.001–0.91)	1.99 (1.23–2.70)	0.18 (0.05–0.31)	0.12 (0.03–0.23)	−2.1 (−8.0–5.1)

### (c) Inbreeding depression in *p*EPO

Across the 204 females, mean *f* was 0.059 (median 0.052, range 0.000–0.305). The posterior mode for the regression on *f* was negative (table 1), suggesting that inbred females tended to produce a smaller proportion of EPO than outbred females (table 1). However, the 95% CI was wide and included substantially positive and negative effects (table 1). Posterior modes for *V*_A,*p*EPO_ and 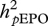 remained quantitatively similar whether or not the regression on *f* was included in the animal model.

## 4. Discussion

Comprehensive understanding of the evolution of extra-pair reproduction in socially monogamous species ultimately requires rigorous estimation of all components of direct and indirect selection acting on males and females [3–5,7]. This task, however, is extremely challenging empirically; key quantities have not been estimated comprehensively or at all and available estimates are often inconsistent, meaning that specific hypotheses can scarcely be rigorously tested or definitive conclusions drawn [4–6].

### (a) Indirect additive genetic benefits

One key hypothesis is that female extra-pair reproduction reflects indirect genetic benefits manifested as increased additive genetic value of offspring (‘good genes’ [1,5,6,8]). The evolution of extra-pair reproduction through such indirect selection requires that the proportion of a female's offspring that is sired by an extra-pair male (*p*EPO) shows additive genetic variance (*V*_A,*p*EPO_ > 0) and is heritable (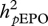 > 0, equation (1.1)), yet these quantities have not been estimated [5]. The heritability of *p*EPO is more relevant than the heritability of female EPC rate in this specific context, since EPCs that do not translate into EPO cannot cause linkage disequilibrium between genes conferring propensity for extra-pair mating and high fitness [5]. In contrast, the heritability of EPC rate is relevant in the context of quantifying certain components of direct selection on extra-pair mating behaviour, since EPCs that do not produce EPO could still impose direct costs (such as sexually transmitted disease [4]). Such estimates of direct and indirect selection could ultimately be connected by quantifying the covariance between EPC rate and *p*EPO [43,44], thereby estimating total selection on EPC behaviour (which underlies EPP). Our analyses of 16 years of comprehensive paternity and pedigree data from socially monogamous but genetically polygynandrous song sparrows showed substantial additive genetic variance (*V*_A,*p*EPO_) and non-zero heritability (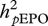) in the proportion of a female's offspring that was sired by an extra-pair male (*p*EPO).

Such *V*_A,*p*EPO_ could reflect *V*_A_ in female EPC rate and/or in the probability of fertilization by extra-pair sperm given EPC. Without data describing female EPC or fertilization rates, which are rare in general [4], we cannot distinguish these possibilities. Furthermore, since paternity was assigned to ringed offspring, the observed *V*_A,*p*EPO_ could conceivably reflect *V*_A_ in differential pre-ringing mortality of EPO versus WPO rather than (solely) *V*_A_ in the proportion of conceived offspring that were EPO. However, except in the specific circumstance that differential pre-ringing mortality of EPO versus WPO was completely compensatory such that mean offspring survival to ringing was constant across females, any such *V*_A_ in differential mortality should be detectable as *V*_A_ in the proportion of eggs laid that survived to ringing. In fact, the egg to ringing survival rate across clutches where at least one offspring survived to ringing averaged 0.80 and varied among females (posterior mode for *V*_I_: 0.34, 95% CI: 0.09–0.57), but showed little detectable additive genetic variance (posterior mode for *V*_A_: 0.001, 95% CI: <0.0001–0.25). The observed *V*_A,*p*EPO_ therefore most probably reflects additive genetic variance in the proportion of conceived offspring that were EPO.

Non-zero 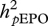 is necessary for indirect additive genetic benefits to contribute to the evolution of female extra-pair reproduction [5], as is heritability of female mating preferences in quantitative genetic models of mate choice evolution more generally [14–16,18]. Our data therefore leave open the potential for such an indirect mechanism to act as widely hypothesized. However, our estimates of 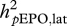 = 0.18 and 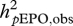 = 0.12, and even the upper 95% credible limits of 0.31 and 0.23, respectively, are lower than the values assumed in published parameterizations of *Δ*_I_ (*h*^2^ = 0.4–1.0 [5]). Multiplying the data scale 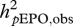 by the overall phenotypic standard deviation of *σ*_*p*EPO_ = 0.32 gives *Δ*_I_ ≈ 0.12.0.32.*d*_EW_ ≈ 0.038.*d*_EW_ (equation (1.1)). Any difference in (additive genetic) fitness between EPO and WPO would therefore need to be relatively large in order to generate rapid evolution of female extra-pair reproduction through indirect additive genetic benefits. Although rigorous estimates of *d*_EW_ are arguably still lacking, available evidence suggests that the phenotypic fitness difference between EPO and WPO may not be large [5,6,13]. Furthermore, the assumption that variation in phenotypic fitness entirely reflects additive genetic variance is unlikely to be correct (e.g. [24,25]), meaning that *Δ*_I_ may be even smaller than estimated from differences in phenotype (or conceivably larger if additive genetic effects were masked by environmental effects). Arnqvist & Kirkpatrick [5] suggest that *Δ*_I_ is likely to be small even given moderate 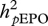. Our evidence that 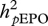 is smaller than previously assumed suggests that *Δ*_I_ is likely to be small even given moderate *d*_EW_, and moreover suggests that any force of indirect selection against extra-pair reproduction, as could arise if *d*_EW_ < 0, is also likely to be small. Indirect selection owing to additive genetic benefits or costs therefore appears unlikely to be a major force driving rapid evolution of female extra-pair reproduction (see also [5,6,16]). Such explicit quantitative conclusions should, however, be drawn given that equation (1.1) assumes normal trait distributions and that additive genetic effects are equally expressed in sons and daughters, which may not be the case. These results do not necessarily preclude the evolution of female extra-pair reproduction through indirect non-additive genetic benefits (‘compatible genes’ [2,8]). Indeed, this mechanism may be more likely given that dominance genetic variance and inbreeding depression are often observed in fitness [27,29,45]. In the absence of such effects, the possibility that female extra-pair reproduction reflects the outcome of sexual conflict [5] remains to be explicitly tested.

### (b) Additional genetic mechanisms

Evidence of substantial *V*_A,*p*EPO_ and non-zero 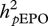 has further interesting implications for mating system evolution beyond solely estimating *Δ*_I_. Notwithstanding constraints imposed by genetic covariation with other traits under selection, non-zero 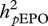 implies the potential for a continued evolutionary response to selection on female EPP rate. Furthermore, the substantial *V*_A,*p*EPO_ implies that, given *V*_A_ in male extra-pair reproductive success, female and male extra-pair reproduction could become genetically correlated, potentially allowing evolution of extra-pair reproduction analogous to that hypothesized to underlie ornamental secondary sexual traits and sperm competitiveness [15,17,18,46]. This possibility requires further explicit consideration. Moreover, direct selection for male social mate choice for females that are less likely to have EPO, and hence covariance between genes underlying such a male preference and female *p*EPO, might also be hypothesized. Finally, non-zero 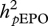 implies that different maternal lineages will comprise different proportions of full-sibs versus maternal half-sibs, potentially causing among-lineage variation in the potential for first-order inbreeding and kin selection. Our evidence of substantial *V*_A,*p*EPO_ and non-zero 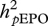 therefore allows for multiple genetic mechanisms to drive and constrain mating system evolution.
